# Predictors in the treatment of malignant central airway obstruction with silicone stents

**DOI:** 10.1007/s00405-023-08365-3

**Published:** 2024-01-06

**Authors:** Mads Bøgh, Søren Gade, Dalia Gustaityté Larsen, Sten Schytte, Ulrik Pedersen, Thomas Kjærgaard

**Affiliations:** 1 Otorhinolaryngology, Head and Neck Surgery, Aarhus University Hospital, Aarhus, Denmark; 2 Otorhinolaryngology, Head and Neck Surgery, Aalborg University Hospital, Aalborg, Denmark

**Keywords:** Stent, Endoscopic treatment, Central airway obstruction, Malignancy

## Abstract

**Purpose:**

To examine the role of the silicone stent in palliation of malignant central airway obstruction and identify potential preprocedural predictors for postprocedural outcome.

**Methods:**

Patients treated with endoscopic insertion of tracheobronchial silicone stents for malignant central airway obstruction at Aarhus University Hospital from 2012 to 2022 were identified from electronic medical records. Statistical analyses were carried out to identify factors affecting Days Alive and Out of Hospital, complications and overall survival.

**Results:**

81 patients underwent a total of 90 tracheobronchial stent insertions. Days Alive and Out of Hospital (DAOH) for the first 30 days were affected negatively by urgent intervention, *p* < 0.001, preprocedural non-invasive respiratory support, *p* < 0.001, and preprocedural intubation, *p* = 0.02. Post-procedural oncological treatment was associated with a significant improved DAOH, *p* = 0.04. Symptomatology and lesion characteristics were not significantly associated with any impact on DAOH. Overall survival was poor (mean survival was 158 days), and only significantly affected by severe degree of dyspnea, *p* = 0.02, and postprocedural oncological treatment, *p* < 0.001. Complication where registered in 25.6% of cases within the first 30 days was observed. Procedure-related mortality was 3.7%. Based on chart annotations by an ENT-surgeon, 95% of the patients experienced relief of symptoms following stent insertion.

**Conclusions:**

Palliative tracheobronchial airway stenting with silicone stents is found to have a beneficial impact, more research is required for identification of predictors for postprocedural outcome based on preprocedural classifications.

## Introduction

Tracheobronchial stents can be endoscopically inserted for immediate restoration of airway patency and palliation of respiratory symptoms, thus improving quality of life in patients with malignant central airway obstruction or stenosis (CAO) [1–3].

Defined as narrowing of the trachea and main stem bronchi, CAO may present with symptoms ranging from mild dyspnea on exertion through severe respiratory insufficiency to potentially lethal respiratory failure [4, 5]. CAO develops secondary to multiple causes in patients of all ages. The most common cause is thoracic malignancy, while the non-malignant causes include benign neoplasms, non-neoplastic diseases and iatrogenic causes. Thoracic malignancies may cause extrinsic compression or intraluminal invasion of the central airways; the most common malignancy to cause CAO is lung cancer [6–9].

Due to the challenge posed by placing a static prosthesis in a dynamic airway, palliative airway stenting in patients with malignant CAO is highly specialised. Both during and after the procedure, airway stenting, which is often combined with endoscopic tumour debulking, is associated with potential life threatening risks in the form of bleeding, mediastinitis, stent rupture, migration or misplacement, and airway obstruction caused by the stent itself [10, 11].

Recent studies have, therefore, examined possible predictors for best outcomes, rate of complications and overall survival after palliative stenting in malignant CAO. However, most of these studies include only a small population of patients stented with silicone stents in comparison to patients stented with self-expandable metallic stents (SEMS) [11–16], thus limiting their generalisability to the former population.

Therefore, the aim of this study is to examine the role of the silicone stent in palliation of malignant CAO in a larger population of different types of thoracic malignancies. Furthermore, this study will apply standardised classifications, such as the Freitag classification [17] and the American Society of Anaesthesiologists Physical (ASA) Status Classification System as possible predictors for outcome after airway stenting. To our knowledge, this will be the first study on tracheobronchial stenting in a Danish population.

## Methods

### Patient population

A retrospective analysis was performed of all patients with malignant CAO who underwent endoscopic tracheobronchial stenting at Aarhus University Hospital, Denmark from 2012 to 2022. Patients who underwent endoscopic tracheobronchial stenting prior to 2012/01/01 were excluded from the analysis. A total of 81 patients received 90 tracheobronchial stents; 73 patients received one stent, 7 patients received 2 stents and 1 patient received 3 stents.

Relevant data were retrieved from the Electronic Medical Record. These data include demographic factors, characteristics of the disease and lesion, additional oncological treatment, urgency of intervention, symptomatology and respiration support, i.e., use of continuous positive airway pressure (CPAP)**/**non-invasive ventilation (NIV), oxygen therapy or intubation. Characteristics of the lesions were classified using the Freitag classification [17]. The patients’ preprocedural condition was classified using the ASA score.

Complications within the first 30 days from intervention were registered, i.e., during the procedure, during the primary admission or following discharge. Complications were classified using the Clavien–Dindo classification [18].

Days Alive and Out of Hospital [19–21] were registered for the first 30 days (DAOH_30_) after stent insertion. If a patient died within the first 30 days after surgery, regardless of whether this was while hospitalised or after they were discharged, the patient was noted for 0 DAOH_30_.

Patient data were anonymously handled in accordance with regional research permit (1-45-70-16-23).

### Airway stents

The stents used were Dumon silicone Stents (Novatech, La Ciotat, France). A Y-stent was inserted if the lesion involved the lower trachea or carina and one or both of the main stem bronchi. Stents were placed with a rigid bronchoscope under general anaesthesia.

### Statistical analysis

Patient data were registered using the REDCap data tool (Aarhus University) and entered into R software. For each patient characteristic shown in Table 1, an unadjusted mean of DAOH_30_ with 95% confidence interval (CI) was calculated. Means of DAOH_30_ were compared using the Welch two-sample *t* test.

**Table 1:** Patient characteristics, procedural details and association with DAOH_30_

	Patient cohort		DAOH_30_	
	*n*	%	Mean (95% CI)	*P* value
Age				
< 65 years	35	43.2%	18.0 (14.1–22.0)	Reference
$\ge$ 65 years	46	56.8%	18.9 (15.6–22.2)	1.0
Gender				
Male	38	46.9%	21.3 (18.1–24.6)	Reference
Female	43	53.1%	**16.0 (12.3–19.7)**	** < 0.001**
Malignancy				
Lung cancer	55	67.9%	19.1 (16.2–22.0)	Reference
Oesophagus cancer	9	11.1%	11.4 (0.9–22.0)	0.06
Other cancers	17	21.0%	20.4 (14.8–26.0)	0.9
Urgency of intervention				
Elective	18	22.2%	21.7 (15.8–27.5)	Reference
Semi-urgent	42	51.9%	19.4 (16.0–22.8)	0.1
Urgent	21	25.9%	**14.1 (9.1–19.1)**	** < 0.001**
Respiratory status				
No respiratory support	42	51.9%	22.2 (19.3–25.1)	Reference
Respiratory support	31	38.3%	**14.6 (10.2–19.1)**	** < 0.001**
Intubated	8	9.9%	**14.1 (3.9–24.4)**	**0.02**
Degree of dyspnea				
None	10	12.3%	24.4 (20.4–28.4)	Reference
Mild–moderate	32	39.5%	18.7 (14.3–23.0)	0.09
Severe	36	44.4%	16.7 (13.0–20.5)	0.5
Unknown	3	3.7%	NA	-
Oncological treatment after stenting				
None	28	34.6%	13.5 (8.5–18.5)	Reference
Yes	51	63.0%	**21.5 (19.0–24.1)**	**0.04**
Unknown	2	2.5%	NA	–
Lesion involving trachea				
No	41	50.6%	20.0 (16.5–23.4)	Reference
Yes	40	49.4%	17.0 (13.4–20.7)	0.2
Lesion involving bronchi				
No	17	21.0%	17.5 (11.3–23.7)	Reference
Yes	64	79.0%	18.8 (16.0–21.6)	0.9
Lesion involving carina				
No	67	82.7%	18.7 (15.9–21.4)	Reference
Yes	14	17.3%	17.9 (10.7–25.0)	0.7
Type of stenosis^a^				
Type 1: Intraluminal	62	75.0%	18.7 (15.9–21.6)	Reference
Type 2: Compression	19	25.0%	19.2 (14.0–24.4)	0.4
Number of affected airway segments				
1	35	43.2%	19.2 (15.5–22.9)	Reference
2	26	32.1%	18.9 (14.2–23.6)	0.9
$\ge$ 3	20	24.7%	16.8 (11.1–22.5)	0.09

*NA* not applicable

^a^Using the Freitag Classification

The Kaplan–Meier method was used for calculation of unadjusted survival. Survival curves were compared using the log-rank test. A *p* value < 0.05 was considered to be statistically significant.

### Outcomes

While short-term prognosis, registered in the form of DAOH_30,_ is the main outcome of this study, the secondary outcomes are short-term complications graded using the Clavien–Dindo classification and survival rates within the first year after stent insertion. Immediate respiratory palliation was assessed within the first 24 h after the procedure by the attending surgeon and based on improvement of dyspnea and respiratory support status.

## Results

### Patient characteristics

Clinical characteristics, procedural details and association with DAOH_30_ are shown in Table 1.

Of the 55 patients with lung cancer, four had small cell carcinoma and 51 had non-small cell carcinoma. Of the 17 patients with other cancers, one had thyroid cancer, one had lymphoma, one had laryngeal cancer, five sarcomas, two had invasive breast cancer and five had metastases from other cancers (prostate cancer (two patients), malignant ameloblastoma, anal cancer and endometrial cancer). Two patients had an unclassified thoracic malignancy at the time of their death.

Of the patients receiving respiratory support at time of intervention, 29 received oxygen therapy, while two received therapy in the form of CPAP/NIV. Eight patients were intubated at time of intervention.

At preprocedural examination and endoscopy, four patients (4.9%) and seven patients (8.6%) were diagnosed with paresis of right and left recurrent laryngeal nerve, respectively. Furthermore, 35 patients (43.2%) had stridor and 20 patients had tachypnea (24.7%). 10 patients reported to have experienced haemoptysis (12.3%), 27 patients reported chronic coughing (33.3%) and 17 patients (21.0%) reported to have experienced recurring pneumonia.

Dyspnea was defined as mild to moderate if the patient experienced dyspnea on exertion, with no regard to the level of activity or work needed to provoke this, and severe if the patient experienced dyspnea at rest.

Intervention was classified as urgent if the procedure was requested and took place within 24 h; semi-urgent, if the procedure took place within 7 days; And elective, if the procedure had been planned for more than 7 days.

In the preprocedural classification of the patient’s condition, only four patients (4.9%) were classified as ASA II, as the majority were classified as ASA III, *n* = 31 (38.3%), or ASA IV, *n* = 44 (54.3%). No patients were classified as ASA I or ASA V; two patients were unclassified at time of intervention. No significant difference in association with DAOH_30_ was found between the patients classified as ASA III and those classified as ASA IV, *p* = 0.7.

### Days alive and out of hospital

DAOH during the initial 30 days after intervention were affected negatively by urgent intervention, *p* < 0.001, preprocedural respiratory support, *p* < 0.001, and preprocedural intubation, *p* = 0.02. Only post-procedural oncological treatment was associated with a significant increase, *p* = 0.04, Table 1.

### Oncological treatment

Only eight patients (9.9%) received no oncological treatment prior to nor following stent insertion. Pre procedural oncological treatment was not associated with any impact on DAOH_30_. Of the 51 patients who received postprocedural oncological treatment, 32 patients (39.5%) received oncological treatment both pre- and postprocedurally.

Immunotherapy in particular was associated with an increase in DAOH_30_, *p* = 0.001, when administered after insertion of the airway stent.

### Complications

Periprocedural complications are shown in Table 2. These were scored using the Clavien–Dindo classification [18]. Grade 1 complications represent any treatment course deviant from the normal postoperative course, requiring antiemetics, analgetics, antipyretics or diuretics. Grade 2 complications require pharmacological treatment with drugs other than those allowed for treatment of grade 1 complications. Grade 3 complications require surgical intervention; Grade 3a requires regional/local anaesthesia, while grade 3b requires intervention performed under general anaesthesia. Grade 4 complications are life-threatening, thus requiring management in an intensive care unit. Grade 4a represents dysfunction of a single organ; 4b of multiple organs. Grade 5 complications are fatal.

**Table 2:** Complications associated with Stenting Procedure^a^

	*n*	%^b^
Time of complication		
Per procedure	1	1.2%
During original admission	12	14.8%
After discharge	10	12.3%
Grade of complication		
1	1	1.2%
2	8	9.9%
3a	0	0.0%
3b	10	12.3%
4a	1	1.2%
4b	0	0.0%
5	3	3.7%
Outcome of complication		
Recovered with No sequelae	19	23.5%
Recovered with sequelae	1	1.2%
Fatal	3	3.7%
Type of complication		
Pneumonia/infection	9	11.1%
Stent migration	4	4.9%
Stent occlusion	5	6.2%
Bleeding	4	4.9%
PE/DVT	1	1.2%

*PE/DVT* Pulmonary embolism/deep vein thrombosis

^a^Using the Clavien–Dindo classification

^b^Percentages are of the entire study population

In total, 23 patients (28.3%) experienced periprocedural complications, thus resulting in a complication rate of 23 complications per 90 procedures (25.6%). Procedure-related mortality was 3.7%. None of the patients experienced more than one complication per procedure. None of the patients, who underwent multiple stenting procedures, experienced complications in relation to more than one procedure.

The three patients who suffered fatal complications were all cases of pneumonia.

### Patient survival

The overall prognosis of patients was poor, with 63 patients (77.8%), and 17 patients (21.0%) alive at 30 days and 365 days after stent insertion, respectively. Accordingly, mortality rates for the first month and first year following stent insertion were 22.2% and 79.0%. Mean survival was 5.3 months. Only one patient (1.2%) was alive after 3 years of follow-up.

Severe preprocedural dyspnea was associated with a significantly worse prognosis compared to asymptomatic, *p* = 0.02, but not mild–moderate dyspnea, *p* = 0.2.

Urgent and semi-urgent procedures were not associated with a worse prognosis, *p* = 0.2 and *p* = 0.3, respectively.

**Fig. 1:**
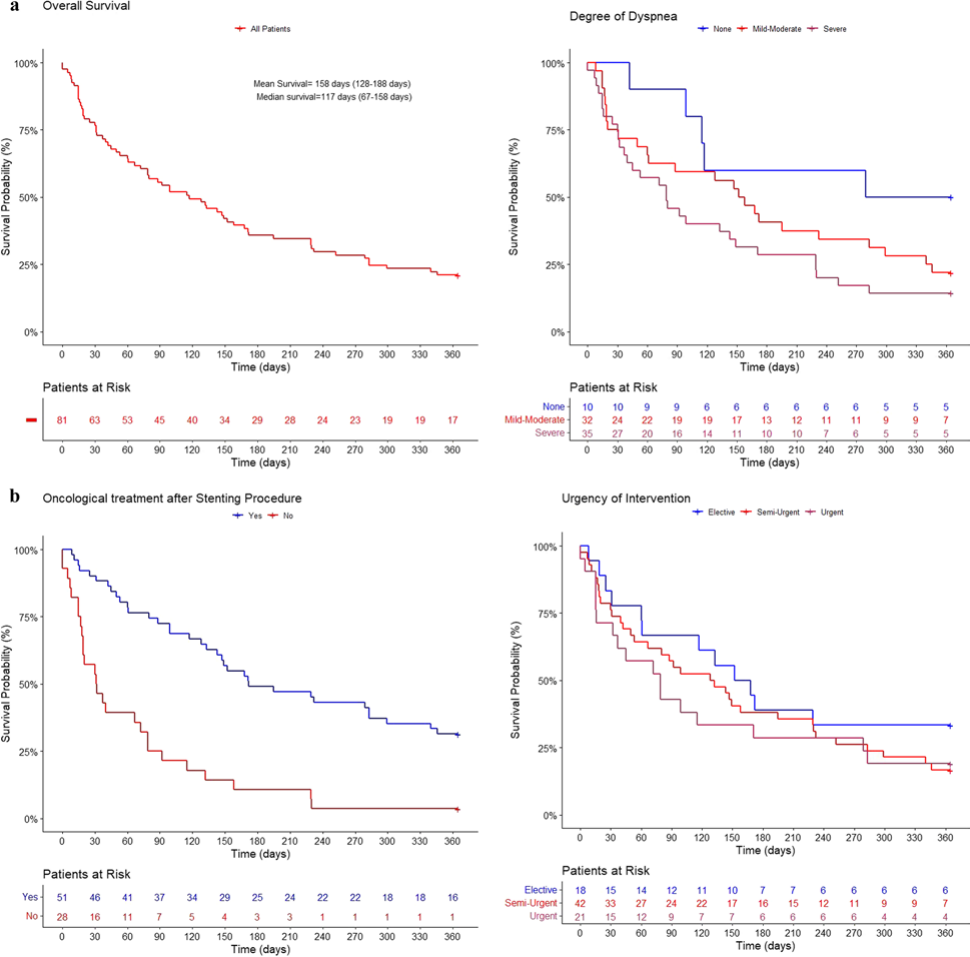
**a** Estimates of overall survival with 95%CI for patients with malignant CAO treated with silicone stents. **b** Severe preprocedural dyspnea was associated with a poor prognosis, *p* = 0.02. **c** Postprocedural oncological treatment was associated with an improvement of prognosis, *p* < 0.001. **d** No significant difference in survival was observed between the three groups of urgency within the first 365 days after stenting

Oncological treatment administered prior to the stent insertion was found to be statistically insignificant in terms of survival, *p* = 0.7, thus mimicking the pattern observed when testing for association with DAOH_30_. However, patients who received oncological treatment after stent insertion, *n* = 19 (23.5%), were found to have a statistically better long-term survival when compared to the patients, who only received oncological treatment prior to stent insertion,* n* = 21 (25.9%), *p* = 0.002. Overall survival was significantly higher in the second half of the inclusion period compared to the first period, *p* = 0.02 (unadjusted analyses, data not shown), paralleling the introduction of biological oncological treatment for lung cancer in Denmark (Fig. 1).

### Immediate respiratory palliation

Seventy-seven of the patients were registered as having an effect of the procedure in terms of respiratory palliation. In two cases, it was not possible to assess the effect of the stent, while it was found to have had a negative if any effect in another two cases. Thus, in 95% of cases, the intervention resulted in relief of symptoms, based on chart annotations by an ENT-surgeon.

## Discussion

Our findings suggest that patients who received oncological treatment after endoscopic stent insertion have a significantly better short-term prognosis in terms of DAOH_30_ as well as long-term survival than patients who only received preprocedural oncological treatment. Thus, in addition to the immediate effect on symptom relief by stent insertion, some degree of disease control was achieved by post-procedural oncological treatment. Although the effect of different palliative oncological regimes was not addressed specifically, our data suggest an additional effect on survival of immunotherapy, which was introduced approximately halfway during the inclusion period. When testing for impact on DAOH_30_, the use of immunotherapy was associated with an improvement of prognosis when administered both prior to and after stent insertion.

Our results also indicate that respiratory status before stent insertion was a viable predictor for DAOH_30_ following insertion of airway stent, as intubation and respiratory support alike was associated with a poor short-term prognosis. Surprisingly, degree of dyspnea was not found to have any impact on DAOH_30_. Nevertheless, severe dyspnea was associated with a poor prognosis when testing for 1-year survival. As opposed to degree of dyspnea, in terms of urgency of intervention, only urgent interventions were associated with a significant decrease in DAOH_30_, yet not found to have any impact on 1-year survival.

**Fig. 2:**
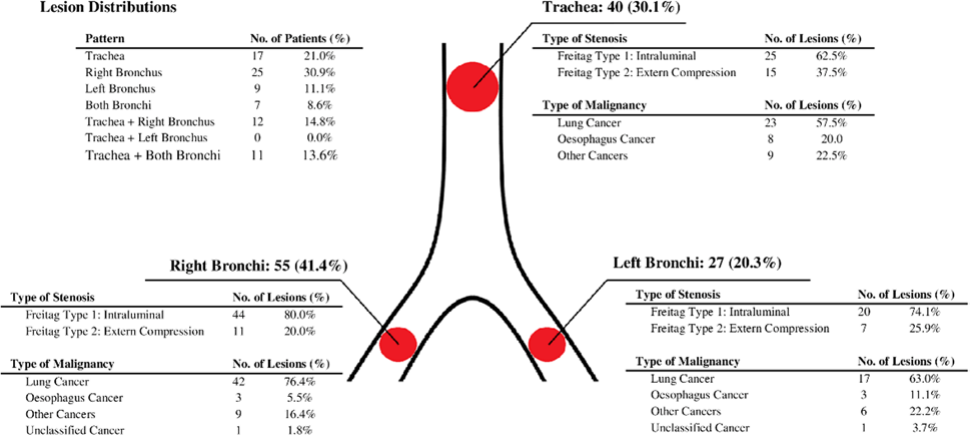
Distribution of lesions in trachea, right and left bronchi. Also shown is the pattern of these lesions, the type of stenosis using the Freitag Classification and type of malignancy

Examining impact on short-term prognosis only, female sex was found to be associated with a significant decrease in DAOH_30_. Furthermore, our findings suggest that neither type of malignancy, site of lesion, type of stenosis according to the Freitag classification nor number of affected airway segments was associated with any impact on DAOH_30_. For reference, study by Iyoda et al. in 2021 found oesophageal cancer to be associated with a significantly worse long-term prognosis, though they omit to report of short-term (1 month) prognosis. Furthermore, although the Freitag classification is useful for description of the type of stenosis, it omits any description of tumour size, thus not providing a just description of the overall prognosis (Fig. 2).

Overall survival in patients undergoing endoscopic tracheobronchial stenting was poor, as the 30-day mortality in this study was 22.2%, while 1-year mortality was 79.0%. Mean survival was 5.3 months. This estimation is slightly lower than what is found in other studies, as the aforementioned study by Iyoda et al. in 2021 [11], comparing overall survival in patients with thoracic malignancy stented with SEMS or silicone stents, reported a median survival of 5.585 months in patients treated with silicone stents. Concurrently, Iyoda et al. reports of a lower 30-day (13.3%) and 1-year mortality (25.1%) as well. A study by Sökücü et al. in 2018 [13] reported a mean survival of 164.51 ($\pm$ 38.83) days, while a study by Lachkar et al. in 2020 [12] reported a median survival of 171 days when examining a similar population treated with silicone Y-stents. Our findings, therefore, match those of Sökücü et al., albeit not those of Lachkar et al.

The 30-day complication rate in our study was 25.6%. A study by Ortiz-Comino et al. in 2021[14] reported a complication rate of 36.7% in the first month following the procedure. However, apart from only including patients symptomatic at time of intervention, their most common complication was mucus retention (75.9%), whereas we found the most common complication the be pneumonia (39.1% of all complications).

No study on endoscopic stenting using silicone stents has examined immediate symptomatic palliation based on improvement of dyspnea or need of respiratory support.

The limitations of this study were multiple. First, it was a retrospective study. Second, though this study comprised the biggest population of patients stented with silicone stents to our knowledge, only 81 patients were eligible for inclusion. Third, as a result of the time span of the inclusion period, changes in oncological treatment regimes, i.e., the introduction of immunotherapy, may have altered the oncological outcome. Subanalyses indeed indicated a significantly better overall survival in the second half of the inclusion period compared to the first period, paralleling the introduction of immunotherapy. Differences in experience level amongst surgeons may also have influenced outcomes, although the majority of interventions were carried out by one surgeon (last author).

## Conclusion

Our findings suggest a positive effect of palliative tracheobronchial airway stenting with silicone stents in patients with thoracic malignancy, though failing to identify any predictors from preprocedural classifications of patient condition. Only urgency of intervention and respiratory status was found to be eligible predictors in terms of DAOH_30_, and DAOH_30_ and overall survival, respectively. More research is, therefore, needed for identification of predictors of postprocedural outcome based on preprocedural classifications.

## Abbreviations

ASA: American Society of Anaesthesiologists
CAO: Central airway obstruction
CI: Confidence interval
CPAP: Continuous positive airway pressure
DAOH: Days alive and out of hospital
NIV: Non-invasive ventilation
SEMS: Self-expandable metallic stent
